# Impacts of Various Reheating Methods on Crispy Chicken: Physicochemical Properties, Oxidation and Flavor Profiles

**DOI:** 10.3390/foods14091574

**Published:** 2025-04-29

**Authors:** Xiaona Ren, Chun Wang, Xueqing Wang, Tingting Su, Yigang Yu

**Affiliations:** 1 The College of Life and Geographic Sciences, Kashi University, Kashi 844000, China; renxiaona1117@163.com (X.R.); 15294178620@163.com (X.W.); sutt0217@163.com (T.S.); 2 School of Food Science and Engineering, South China University of Technology, Guangzhou 510640, China; wang05212000@163.com

**Keywords:** crispy chicken, reheating methods, physicochemical properties, oxidation, flavor characteristics

## Abstract

In this study, the impacts of water-bath reheating (WR), steam reheating (SR), air-frying reheating (AR), roasting reheating (RR), and microwave reheating (MR) on the physicochemical properties, oxidation, and flavor profiles of crispy chicken (CC) were investigated. The results revealed that the pH of CC was significantly reduced after reheating (*p* < 0.05). The AR samples had a slight change in L* and the highest springiness. The RR samples had the highest degree of lipid and protein oxidation. In addition, WR, AR, RR, and MR treatments effectively increased the contents of umami-related amino acids. Glu and Cys were typically the taste-active amino acids in CC. AR contributed to increasing the response values of umami and richness. As shown by the electronic nose and Gas Chromatography–Mass Spectrometry (GC-MS) analysis, 41 volatile compounds were obtained in CC. AR could efficiently increase the contents of nitrogen oxides and methyl compounds. Meanwhile, the content of trans-.alpha.-bergamotene, nonanal, and copaene were significantly increased after the AR process (*p* < 0.05). According to the results of analysis of variance (ANOVA), odor activity value (OAV), and variable importance in projection (VIP), anethole was considered the key differential flavor-active compound. Overall, AR was superior to other reheating methods in CC, with better texture and various flavor characteristics. This study provides a reference for choosing reheating technology for pre-cooked chicken products.

## 1. Introduction

Crispy chicken (CC), a kind of fried product, is one of the Chinese traditional dishes. The production processes of CC include seasoning, marinating, hanging flour, and frying [1]. The purchase requirement for consumers gradually rises owing to its crispy texture, high tenderness, and desirable color [2]. In recent years, consumers have been more likely to buy pre-cooked food products due to the fast-paced lifestyle and evolving dietary preferences [3]. Consequently, pre-cooked food products with various flavors and packages have been developed to satisfy diverse consumer demands. The CC products are mainly sold in supermarkets and online in a frozen, pre-cooked form [4]. They have advantages such as convenient storage, extended shelf life, and ready-to-eat convenience upon heating. Based on the advantages above, they have a high market share and are widely used in both the catering industry and household meals [5].

Reheating is a critical part of pre-cooked food, which is very important to improve the edible qualities such as appearance, taste, and flavors. For pre-cooked frozen food, heating to at least 70 °C for 1 min can inhibit the growth of most microorganisms [6]. The common reheating methods are mainly water bath reheating, steam reheating, and microwave reheating [7]. Water-bath reheating is a method of uniform heat transfer. The food is shielded from direct air exposure in this process, reducing moisture loss and textural changes due to uneven heating [8]. However, the low temperature can lead to reduced heating efficiency [9]. Steam reheating is a process that uses water vapor as a medium to transfer heat to the food. This method can reduce water loss, but prolonged heating stimulates a soft texture in the food [10]. Water molecules vibrate rapidly in the microwave field, generating heat to reheat the food. Microwave reheating reduces the nutrient loss of food, with low energy consumption and a high heating rate [11].

However, roasting reheating is also an option for fried foods. Roasting is a method in which food is heated through hot air and metal pans (convection and conduction). Nevertheless, excessive temperature and time may result in the generation of polycyclic aromatic hydrocarbons and heterocyclic amines, leading to fat malabsorption and reduced kidney size [12]. Air-frying technology was introduced from Europe as a new heating technology. This method is achieved through the direct contact between a fine mist of oil droplets in hot air and the food inside a container [13]. Studies have shown that air-fried food has a higher nutritional quality than with conventional frying, which possibly was related to reduced lipid degradation and oxidation. The acrylamide content was decreased by approximately 90% [14]. The outer shell of the food is crisper and thinner, with uniform thickness due to less starch pasting [15]. Thus, air-frying reheating is an alternative method for healthier fried food. Recently, researchers have focused mainly on the effects of different thermal treatments and parameters on the oxidation, sensory evaluation, moisture migration, and flavor profiles of meat. There are fewer studies on the changes in the quality of pre-cooked food (especially fried meat) after air-frying reheating. Therefore, it is vital for us to evaluate the impacts of various reheating methods on CC from all angles.

In this study, the effects of water-bath reheating (WR), steam reheating (SR), air-frying reheating (AR), roasting reheating (RR), and microwave reheating (MR) on the physicochemical properties (pH, color, and texture) of CC were evaluated. Meanwhile, the lipid oxidation and protein oxidation were illustrated to describe the quality of the CC. In addition, the taste characteristics were identified according to the analysis of electronic tongue (E-tongue), free amino acid (FAA), and taste activity value (TAV). The volatile compounds were identified by tests with the electronic nose (E-nose) and GC-MS. The study provides a theoretical basis for the reheating processing of pre-cooked chicken products.

## 2. Materials and Methods

### 2.1. Materials

Whole chicken breasts and spices were chosen and purchased from Guangzhou Xiangxian Technology Co., Ltd. (Guangzhou, China). Whole chicken breasts were divided and processed within 2 h, and then transported to the laboratory (stored at 4 °C). The muscle part of the chicken breasts was the pectoralis major. 3-Heptanone, 2-methyl- was purchased from Sigma-Aldrich (St. Louis, MO, USA). Other chemicals (AR grade) were purchased from Shanghai Aladdin Biochemical Technology Co., Ltd. (Shanghai, China).

### 2.2. Samples

#### 2.2.1. Preparation of CC

CC was produced according to the method of Ananey-Obiri et al. [1]. Chicken breasts were cut into strips (5 × 1 × 1 cm^3^), and marinated in cooking wine, soy sauce, salt, chicken essence, thirteen spice, and turmeric powder (at a mass ratio of 1:0.2:0.3:0.16:0.04:0.06) for 15 min at 4 °C. Then, the samples were immersed in paste for 15 s. The paste consisted of medium-gluten flour, potato starch, and water at a mass ratio of 1:1:2. After that, the samples were fried at 149 °C for 40 s and then fried at 179 °C for 107 s in the peanut oil.

#### 2.2.2. Sample Processing

The CC was stored in a freezer at −18 °C (DW-25L262, Haier Co., Ltd., Qingdao, China) for 7 d, and then thawed at 4 °C until the central temperature reached 4 °C. According to the preliminary experiments, the thawed samples were randomly divided into 6 groups for different reheating treatments. (1) CK: non-reheating treatment. (2) Water-bath reheating (WR): the samples were placed in sealed bags (150 × 150 mm^2^, Wuhu Xiangkou Household Products Co., Ltd., Wuhu, China) and reheated in boiling water (100 °C). (3) Steam reheating (SR): some boiling water was added to a steamer (ZGB2605, Joyoung Co., Ltd., Jinan, China), and the samples were placed a pan with small holes above the boiling water and reheated (100 °C). (4) Air-frying reheating (AR): the samples were reheated in an air fryer (HD9100/80, PHILIPS Co., Ltd., Shanghai, China) at 180 °C. (5) Roasting reheating (RR): the samples were reheated in an oven (PT3540, Midea Co., Ltd., Foshan, China) at 180 °C, and the RR process was heated by a combination of convection and conduction. (6) Microwave reheating (MR): the samples were reheated in a microwave oven (PM2000, Midea Co., Ltd., Foshan, China) at a power of 500 W. All the samples were reheated until the center temperature was 75 °C and then cooled to 25 °C for analysis.

### 2.3. pH

pH was measured according to the method of Jo et al. [16]: 10 g of the samples was mixed with 90 mL of deionized water and placed in a refrigerator at 4 °C for 30 min. The pH was measured through a portable pH meter (PH838, SMART Co., Ltd., Dongguan, China). The type of pH probe was electrode probe (E-201F, Shanghai Instrument & Electrical Scientific Instrument Co., Ltd., Shanghai, China).

### 2.4. Color

Color was measured using a portable colorimeter (Ci60, X-rite Color Management Co., Ltd., Shanghai, China). The parameters were set as follows: light source of D65, observer of 10°, and measurement mode of QA. The L*, a*, and b* values were determined at random positions on the surface of the samples. Each analysis was repeated 5 times for each treatment.

### 2.5. Texture Profile Analysis

The texture profile analysis (TPA) was investigated using a Texture Analyzer (TMS-PRO, FTC Co., Ltd., Sterling, VA, USA) according to Li et al. [17]. The parameters were set as follows: disc probe with a diameter of 75 mm, force-sensing element of 100 N, constant speeds of 0.5 mm/s (pre-test, mid-test, and post-test), a compression ratio of 50%, a starting force of 0.15 N, and a dwell time of 5 s. Each analysis was repeated 5 times for each treatment.

### 2.6. Lipid Oxidation

Lipid oxidation is usually expressed as the thiobarbituric acid reactive substance (TBARS) value according to the method of Wang et al. [18]. First, 2 g of the samples was blended with 3 mL of TBA (1%, *w*/*v*), 17 mL of TCA-HCl (2.5%, *v*/*v*), and 0.1 mL of butylated hydroxytoluene (0.01%, *w*/*v*). Then, the mixture was heated in the boiling water for 30 min. After cooling, the mixture was centrifuged at 8000 r/min for 10 min in a centrifuge (H1750R, Xiangyi Testing Equipment Co., Ltd., Changsha, China). The absorbance of the supernatant was recorded at 532 nm. The TBARS value was expressed as a malondialdehyde (MDA) content. The formula was as follows:

(1) $$\mathrm{TBARS}\;(\mathrm{mg}\;\mathrm{MDA}/\mathrm{kg})=\frac{A_{532}}{m_{0}}\text{×}9.48$$

where *A*_532_ is the absorbance of the supernatant at 532 nm, *m*_0_ is the weight of the sample (g), and 9.48 is a derived constant from the dilution factor and the molar extinction coefficient (152,000 M^−1^ cm^−1^) of the TBA reaction product.

### 2.7. Protein Oxidation

#### 2.7.1. Carbonyl Content

The protein solution of CC was obtained by the method of Jiang et al. [19]. Briefly, 1 g of the samples was added to 10 mL of PBS solution (0.1 mol/L, pH 7.2). Then, the mixture was homogenized at 10,000 r/min for 30 s and centrifuged at 8000 r/min for 10 min. The supernatant was the protein solution. The carbonyl content of the protein solution was evaluated following the method of Chen et al. [20] using DNPH. After the reaction, the absorbance of the solution was recorded at 370 nm. The carbonyl content (nmol/mg protein) was calculated using the absorbance coefficient of 22,000 M^−1^·cm^−1^.

#### 2.7.2. Total Sulfhydryl Content

The total sulfhydryl content of the protein solution was expressed by the method of Zhang et al. [21]. First, 1 mL of the protein solution was mixed with 9 mL of 0.2 mol/L Tris-HCl (8 mol/L urea, 2% SDS (*w*/*v*), 10 mmol/L EDTA, pH 6.8), and 1 mL of 0.1% DTNB (*w*/*v*, pH 8.0). Then, the mixture was reacted for 25 min in the water bath (40 °C). After that, the absorbance of the solution was measured at 412 nm. The result (nmol/mg protein) was calculated using the absorbance coefficient of 13,600 M^−1^·cm^−1^.

### 2.8. E-Tongue Analysis

E-tongue analysis system (TS-5000Z, Insent Co., Ltd., Tokyo, Japan) was used to analyze the tastes of CC by the method of Cheng et al. [22]. The samples (10 g) and deionized water (100 mL) were homogenized at 5000 r/min for 1 min, then placed for 30 min (4 °C), and the supernatant (80 mL) was obtained for analysis after being filtered.

### 2.9. Free Amino Acid (FAA) and Taste Activity Value (TAV) Analysis

FAA was determined according to the method of Qi et al. [23]. The samples (2 g) were added to 10 mL of sulfosalicylic acid solution (10%, *w*/*v*). The mixture was homogenized at 7000 r/min for 40 s, then centrifuged at 10,000 r/min for 15 min (4 °C), and the supernatant was obtained for analysis. Then, 4 mL of the supernatant and 4 mL of petroleum ether were mixed thoroughly, and the petroleum ether was removed. After that, 1 mL of the supernatant was diluted 10-fold, filtered with 0.22 µm aqueous filtration membrane, and analyzed using an Amino Acid Automatic Analyzer (LA-8080, Hitachi Co., Ltd., Tokyo, Japan). TAV was calculated as the ratio of the concentration of FAA (mg/g) to its threshold value (mg/g) by the method of Fan et al. [24].

### 2.10. E-Nose Analysis

E-nose analysis was performed according to Chen et al. [25]. First, 3 g of the samples was placed in a 20 mL headspace bottle and kept at 50 °C for 30 min. Then, the samples were analyzed using an E-nose analysis system (PEN3, Airsense Co., Ltd., Schwerin, Germany). The parameters were set as follows: sample preparation time of 5 s and determination time of 1 min. Ten sensors and their flavor characteristics are shown in Table 1.

### 2.11. GC-MS Analysis

The volatile compounds (VOCs) were measured according to the method of Sun et al. [26] with slight modifications using a GC-MS device (7890/5977B, Agilent Technologies Inc., Santa Clara, CA, USA). First, 2 g of the samples was weighed into a 20 mL headspace vial for analysis. The VOCs were extracted with an SPME fiber tip at 55 °C for 30 min, and then held for 10 min. The HP-5 capillary column (30 m × 0.32 mm × 0.25 µm) was utilized to measure the VOCs. The temperature was 40 °C initially, raised to 150 °C (5 °C/min), and then equilibrated for 12 min. Finally, the temperature was increased to 230 °C (8 °C/min) and equilibrated for 3 min. The carrier gas was helium (99.99%), with a flow rate of 1.0 mL/min. The MS parameters were as follows: the scanning range of 30–550 *m*/*z*, the ionization voltage of 70 eV, and the ion source temperature of 230 °C. The VOCs were identified by using the NIST14 mass spectrometry database. The VOCs were semi-quantified using an internal standard consisting of 100 µL of 3-Heptanone, 2-methyl- (0.816 µg/mL). The concentration of VOC was calculated by the following formula:

(2) $$Mi=\frac{Si}{Sb}\times \frac{m}{M}\times 1000$$

where *M_i_* is the concentration of VOC (µg/kg), *S_i_* is the peak area of VOC, *S_b_* is the peak area of 3-Heptanone, 2-methyl-, *m* is the weight of 3-Heptanone, 2-methyl- (µg), and *M* is the weight of CC (g).

The odor activity value (OAV) was obtained by the ratio between the concentration of VOC and its odor threshold. When the OAV was more than 1, this compound was considered as a flavor-active compound [27].

### 2.12. Statistical Analysis

The experimental data of color and texture were measured five times in parallel. The sensory evaluation data were measured ten times in parallel. Other data were measured three times in parallel. The data were expressed as mean ± standard deviation. One-factor analysis of variance and significance (*p* < 0.05) was conducted on SPSS 18.0. Origin 2021 was used for graphing and principal component analysis (PCA). SIMCA 14.1 was used for the orthogonal partial least square discrimination analysis (OPLS-DA). The flavor characteristics of VOCs could be found online at http://www.odour.org.uk.

## 3. Results and Discussion

### 3.1. pH

**Figure 1:**
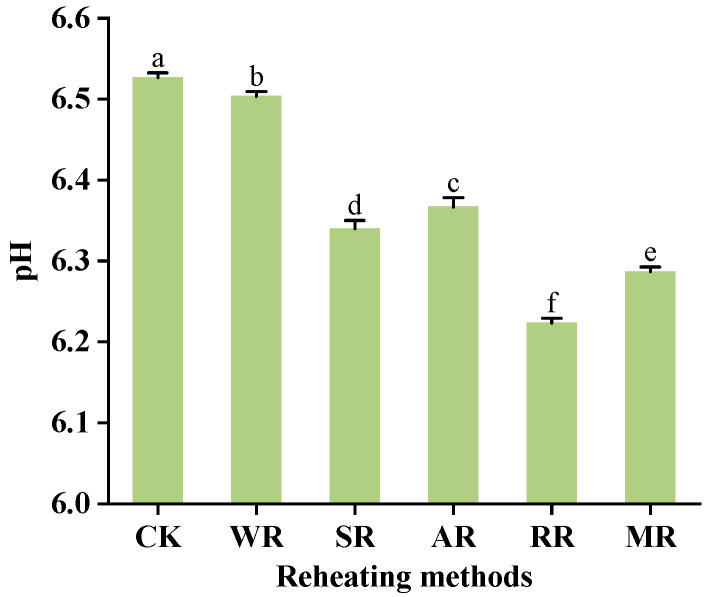
Effects of different reheating methods on pH of CC. CK was non-reheating treatment. WR was water−bath reheating. SR was steam reheating. AR was air−frying reheating. RR was roasting reheating. MR was microwave reheating. a–f mean significant differences between different reheating methods (*p* < 0.05).

As shown in Figure 1, the pH of the samples was significantly reduced (*p* < 0.05) after reheating. This might be attributed to the evaporation of moisture from the CC during the reheating process, leading to the concentration of internal fatty acids and a decrease in pH [28]. The basic amino acids and ammonium salts produced by the oxidation of proteins during heating could cause an increase in pH [29]. Thus, the concentration of fatty acids was identified as the primary reason for the decrease in pH. The pH values of the RR and MR samples were lower (6.22 and 6.29, respectively) than the other samples. The WR samples had a higher pH (6.50). The possible reason was that the rapid moisture evaporation occurred after a period of high-temperature reheating (RR and MR), leading to a fast process of fatty acid concentration. In contrast, the CC was wrapped in the sealing bag and immersed in the boiling water at a lower temperature during the WR process, leading to slow moisture evaporation and a small change in pH.

### 3.2. Color

Color is an important indicator for evaluating the sensory characteristics of meat, which can directly affect the acceptance of meat products among consumers [30]. Table 2 shows that the L* of the SR samples had no significant differences from the CK samples (*p* > 0.05). This might be due to a lower moisture loss, inhibiting light absorption of the surface in the CC [31]. In addition, the L* of the AR samples (68.39) had a little change compared with the CK samples, probably because uniformly distributed hot air was produced during the AR process, facilitating the even distribution of oil droplets and minimizing the contact between the CC surface and oil. As a result, excessive browning was mitigated, and a higher L* was preserved [32]. The a* of the WR, SR, AR, RR, and MR treatments increased by 3.91%, 4.32%, 6.37%, 10.96%, and 7.11%, respectively, compared with the CK group. The increased trend might be owing to the Maillard reaction and lipid oxidation under high-temperature conditions, leading to browning [8]. The b* of the CC slightly increased after reheating treatments. The reason for changes in b* might be that high temperatures could promote the reaction between free radicals from unsaturated fatty acid oxidation and amines in proteins, leading to the formation of yellow pigments [33]. In summary, the AR treatment could preserve the original color of the CC better than the other four treatments.

### 3.3. Texture

**Figure 2:**
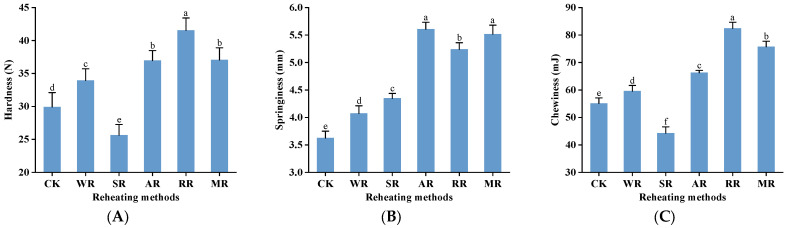
Effects of different reheating methods on hardness (**A**), springiness (**B**), and chewiness (**C**) of CC. CK was non−reheating treatment. WR was water−bath reheating. SR was steam reheating. AR was air−frying reheating. RR was roasting reheating. MR was microwave reheating. a–f mean significant differences between different reheating methods (*p* < 0.05).

Hardness, springiness, and chewiness are critical indicators that determine the texture of meat products. As illustrated in Figure 2A, the hardness of the SR samples decreased by 14.30% compared with the CK group. Studies indicated that a large amount of water absorption occurred after the SR treatment. The muscle fiber structure became expanded and relaxed, resulting in a softer texture of the CC. And the hardness of the samples significantly increased after other treatments (*p* < 0.05) because of moisture loss [34]. Figure 2B indicates that the springiness of the CC increased by 12.37% (WR), 19.95% (SR), 54.70% (AR), 44.61% (RR), and 52.21% (MR), respectively, after reheating. A higher moisture content within the protein network structure facilitated the development of a three-dimensional gel after cooling, which was crucial for maintaining the springiness of the CC [35]. The AR samples had the highest springiness (5.61 mm) among all treatments because the crispy outer shell generated with high-temperature air could retain the moisture inside the CC, promoting the formation of a gel network structure [16]. Figure 2C shows that the chewiness of the CC significantly increased after reheating treatments, except the SR treatment (*p* < 0.05). The RR samples had the highest chewiness (82.43 mJ) among all samples. During the RR process, localized overheating led to more water evaporation. Thus, the toughness of the myofibrillar protein network reduced, and the texture of the CC became hard to chew. These results above illustrated that the AR and MR treatments had better texture among all the samples.

### 3.4. Lipid Oxidation

**Figure 3:**
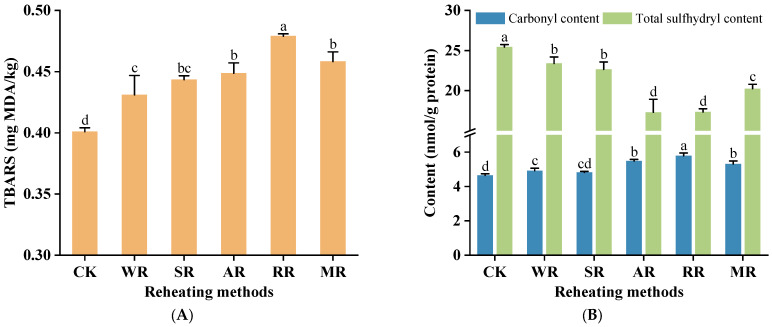
Effects of different reheating methods on TBARS (**A**) and protein oxidation (**B**) of CC. CK was non-reheating treatment. WR was water−bath reheating. SR was steam reheating. AR was air−frying reheating. RR was roasting reheating. MR was microwave reheating. a–d mean significant differences between different reheating methods (*p* < 0.05).

Lipid oxidation usually causes off-flavors, rancidity, and color changes in food. The TBARS value is a common metric used to measure the extent of lipid oxidation in food, particularly in meat products [36]. Figure 3A indicates that the TBARS value significantly increased after reheating (*p* < 0.05). The RR treatment exhibited the highest degree of lipid oxidation (0.479 mg MDA/kg). The oven was operated with a combination of convection and conduction heating [37]. Hot air circulated evenly around the CC, so it was exposed to high-temperature air, enhancing the decomposition of lipids into secondary oxidation products like MDA. Additionally, direct contact with metal components such as baking trays or racks accelerated the heat absorption and transfer to the CC. This rapid reheating process could further increase the rate of lipid oxidation compared with methods where the CC was not in direct contact with hot surfaces. The TBARS value of the WR treatment (0.431 mg MDA/kg) had no significant differences compared with the SR treatment (*p* > 0.05), showing the lowest level. This was possibly due to the low temperature and less oxygen exposure, inhibiting lipid oxidation reactions [38].

### 3.5. Protein Oxidation

#### 3.5.1. Carbonyl Content

Carbonyls mainly refer to oxidation products formed from lysine, proline, arginine, and threonine residues in proteins under free radicals (e.g., OH and O^2−^). The higher the carbonyl content in the protein, the higher the degree of protein oxidation [39]. As shown in Figure 3B, the carbonyl content of the SR samples (4.82 nmol/g protein) showed no significant differences compared with the CK samples (*p* > 0.05). The small change might be because the continuous vapor surrounded the surface of the CC, reducing the contact between amino acid residues and oxygen. Thus, the process could reduce the rate of protein oxidation. However, other treatments showed an increasing trend compared with the CK group, consisting of 5.98% (WR), 18.00% (AR), 24.55% (RR), and 14.35% (MR), respectively. The reason might be that the secondary and tertiary structures of proteins were destroyed during high-temperature or lengthy reheating processes. More reactive sites were exposed, and the reaction between amino acid residues and free radicals was promoted, leading to protein oxidation [29].

#### 3.5.2. Total Sulfhydryl Content

Total sulfhydryl groups included sulfhydryl groups exposed on the surface of the proteins and inside the protein network, which usually existed in sulfur-containing amino acids (e.g., cysteine) [40]. The total sulfhydryl content of the CC was significantly reduced (*p* < 0.05) after reheating (Figure 3B). The total sulfhydryl contents of the WR and SR treatments were at a high level, reduced by 8.04% and 10.94% compared with the CK group. Meanwhile, the total sulfhydryl contents in the AR and RR treatments (17.30 and 17.33 nmol/g protein, respectively) had no significant differences (*p* > 0.05) but were lower than other treatments. It was shown that the tertiary structure of proteins was disrupted under high temperatures. The sulfhydryl groups in the internal network structure of proteins were exposed and oxidated, leading to decreased contents [41]. Therefore, the AR and RR processes (at 180 °C) facilitated the decrease in the total sulfhydryl content. The protein oxidation did not easily proceed during the WR and SR process, with high humidity and low oxygen contents. During the MR process, the heat was generated by friction between water molecules and other polar molecules [42]. This process resulted in less moisture loss and diluting the reactive oxygen species, reducing the sulfhydryl oxidation. Additionally, Wang et al. [43] stated that MDA was able to increase the content of hypervalent myoglobin and non-heme iron, accelerating the protein oxidation in rabbit meat. Therefore, lipid oxidation could also promote protein oxidation.

### 3.6. E-Tongue Analysis

**Figure 4:**
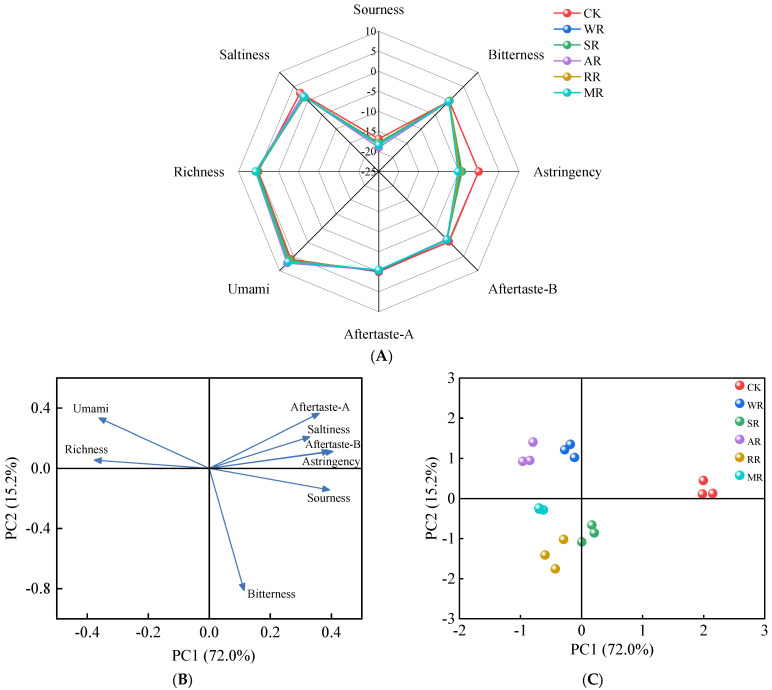
Radar chart (**A**), loading plot (**B**), and score plot (**C**) of E−tongue data for CC with different reheating methods. CK was non−reheating treatment. WR was water−bath reheating. SR was steam reheating. AR was air−frying reheating. RR was roasting reheating. MR was microwave reheating.

The E-tongue can measure taste characteristics of food through a series of sensors by simulating human taste buds [44]. As illustrated in Figure 4A, the taste profiles of CC under different reheating treatments exhibited similarities. The response values of umami, richness, and saltiness were greatly higher than the neutral point, suggesting that they were key taste indicators in the CC [45]. The response values of umami and richness significantly increased after reheating (*p* < 0.05). This increase could be attributed to the production of taste-active peptides through the Maillard reaction and protein degradation [46]. The AR samples had the highest response values of umami and richness, at 7.32 and 5.73, respectively. This might be attributed to the rapid evaporation of water and to lipid solubilization during the AR process, which could concentrate the flavor compounds. Oppositely, the response value of saltiness showed a significant decrease after reheating (*p* < 0.05), with the WR treatment showing the lowest value (1.17). During the WR process, the temperature was relatively low, resulting in less water evaporation. The higher moisture content could dilute the salty substances, thereby reducing the concentration of salty substances and leading to a decrease in the response values of saltiness [47].

Based on the results of PCA, the total variance contribution rate of the two principal components was 87.2%, indicating that the two principal components explained most of the information from the E-tongue. Figure 4B showed that PC2 was negatively correlated with bitterness, and PC1 was positively correlated with astringency, aftertaste-B, and sourness. Figure 4C shows that the CK group was placed on the positive PC1 axis. All reheating treatment groups were placed on the negative PC1 axis and correlated with umami and richness. These results suggested that reheating treatments could increase umami and richness. The RR treatment was related to bitterness, which illustrates that unpleasant taste substances were generated during the prolonged heating process at high temperature.

### 3.7. FAA and TAV Analysis

**Figure 5:**
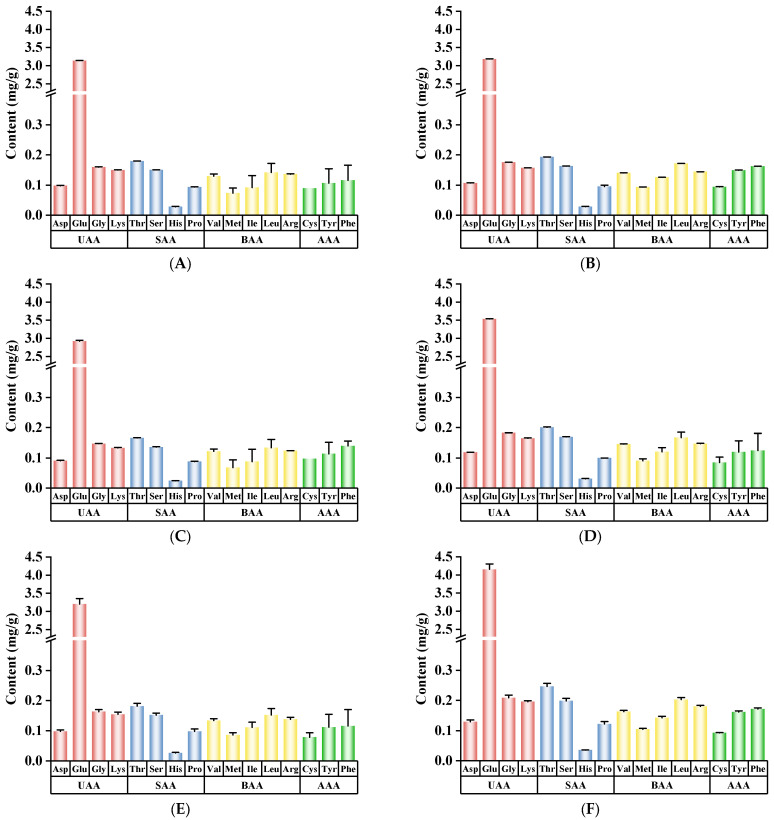
Effects of different reheating methods on FAAs of CC. (**A**–**F**) represent the CK, WR, SR, AR, RR, and MR samples, respectively. CK was non−reheating treatment. WR was water−bath reheating. SR was steam reheating. AR was air−frying reheating. RR was roasting reheating. MR was microwave reheating. (UAA: umami−related amino acid; SAA: sweet−related amino acid; BAA: bitter−related amino acid; AAA: aromatic−related amino acid).

FAAs are the main taste compounds in chicken products [22]. As illustrated in Figure 5, four categories of FAAs were identified in the CC. These included umami-related amino acids (Asp, Glu, Gly, and Lys), sweet-related amino acids (Thr, Ser, His, and Pro), bitter-related amino acids (Val, Met, Ile, Leu, and Arg), and aromatic-related amino acids (Cys, Tyr, and Phe), with umami-related amino acids being the most abundant [48]. The contents of FAAs in CC differed with different reheating methods. The contents of Glu showed a significant increase after the AR and MR treatments (*p* < 0.05). The possible reason was that uniform high-temperature air promoted the hydrolysis of proteins and the release of umami-related amino acids under the Maillard reaction [49]. The sweet-related amino acid contents in the WR, AR, RR, and MR treatments increased by 6.18%, 11.00%, 1.45%, and 33.59%, respectively, compared with the CK group. In contrast, the SR treatment showed a slight decrease. The possible reason was that higher moisture content during the SR process induced hydrolysis reactions in the sweet-related amino acids, thereby leading to a reduction in the content of sweet-related amino acids [50]. In terms of aromatic-related amino acids, the MR treatment increased by 0.12 mg/g compared with the CK group, the highest increase among the five reheating methods.

**Figure 6:**
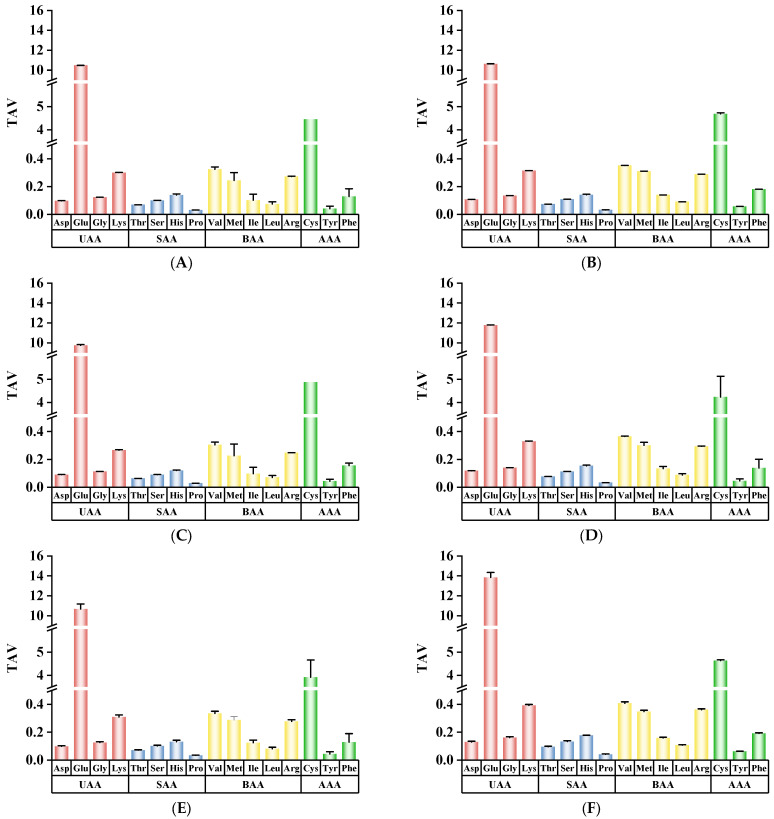
Effects of different reheating methods on TAVs of CC. (**A**–**F**) represent the CK, WR, SR, AR, RR, and MR samples, respectively. CK was non−reheating treatment. WR was water−bath reheating. SR was steam reheating. AR was air−frying reheating. RR was roasting reheating. MR was microwave reheating. (UAA: umami−related amino acid; SAA: sweet−related amino acid; BAA: bitter−related amino acid; AAA: aromatic−related amino acid).

TAV can measure the contribution of a specific amino acid to the overall taste profile. When the TAV is more than 1, this amino acid is thought to contribute significantly to the taste profile of the food [51]. Figure 6 shows that the TAVs of Glu and Cys were greater than 1, indicating that these two amino acids were typically taste-active amino acids in CC. Although the TAVs of the other FAAs were all less than 1, these amino acids still played an important role in the taste function of CC. The TAVs of Glu significantly increased (*p* < 0.05), except in the SR treatment. The MR treatment showed the highest TAV for Glu (13.85). Additionally, the WR, SR, and MR treatments all could increase the TAV of Cys, with the SR treatment having the highest value (4.88).

### 3.8. E-Nose Analysis

**Figure 7:**
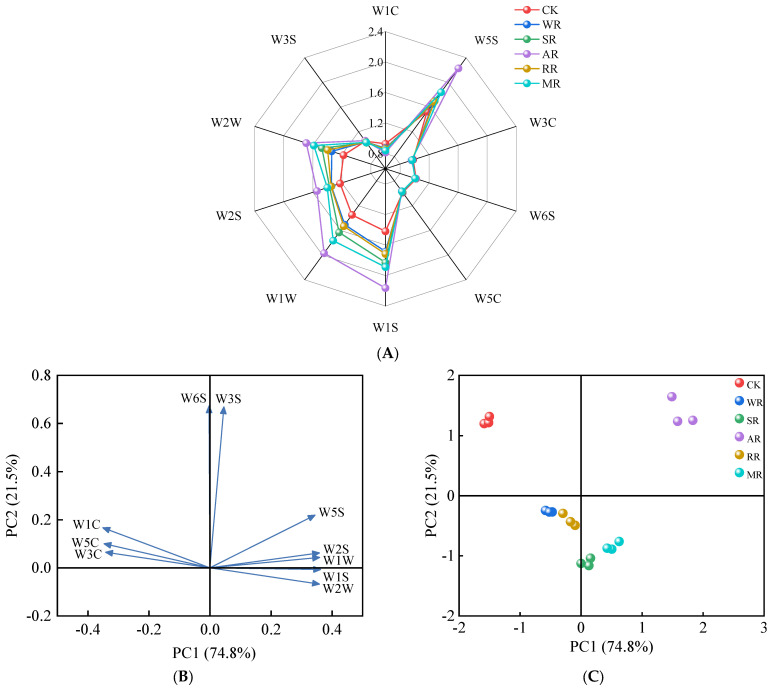
Radar chart (**A**), loading plot (**B**), and score plot (**C**) of E−nose data for CC with different reheating methods. CK was non−reheating treatment. WR was water−bath reheating. SR was steam reheating. AR was air−frying reheating. RR was roasting reheating. MR was microwave reheating.

The E-nose is a technology to simulate the human olfactory system for the detection of VOCs in food, characterized by high sensitivity, non-invasiveness, and rapid response [52]. Figure 7A illustrates that the response values of W5S, W1S, W1W, W2S, and W2W were higher than other sensors in each sample. After reheating, the response values of these five sensors significantly increased (*p* < 0.05). The response value of W5S in the WR, SR, AR, RR, and MR treatments increased by 8.05%, 11.10%, 45.76%, 10.63%, and 20.41%, respectively. The response values of W3C, W6S, W5C, and W3S had no significant difference (*p* > 0.05) compared with CK. The results indicated that reheating treatment could efficiently increase the contents of nitrogen oxides, methyl compounds, inorganic sulfides, alcohols, and organic sulfides in CC. Among them, the AR treatment showed the greatest increase. This might be because the samples were fully in contact with their own fats under convective heating, which promoted lipid oxidation and produced more VOCs [32].

The results of PCA indicated that the cumulative variance contribution rates of PC1 (74.8%) and PC2 (21.5%) were 96.3% (Figure 7B). This suggested that the two principal components could explain most of the flavor characteristics in the CC. The response value of W1S contributed the most to PC1, and the response value of W6S contributed the most to PC2. In addition, the response value of W5S had some influences on both principal components. The AR and MR samples are situated on the positive PC1 axis (Figure 7C), indicating that the two samples had similar flavors. Combined with the radar chart, it can be seen that the AR and MR samples had a higher level of contents of VOCs.

### 3.9. GC-MS Analysis

**Figure 8:**
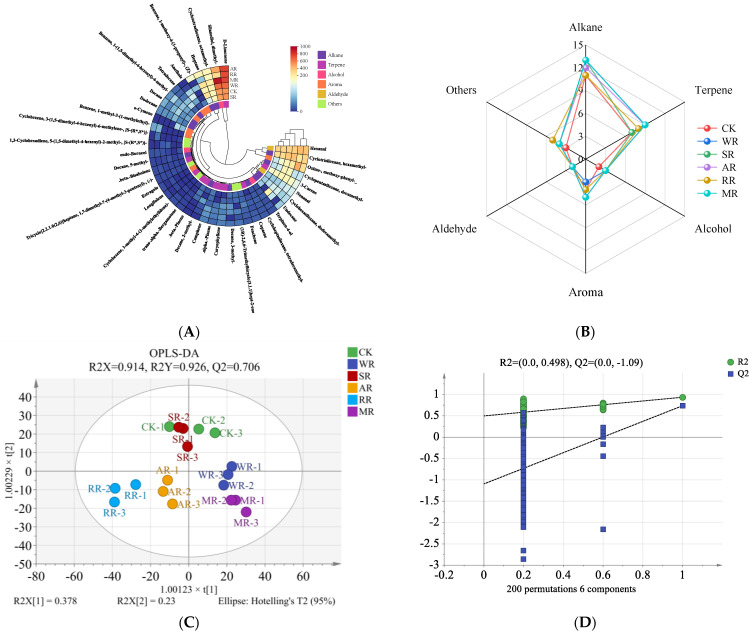

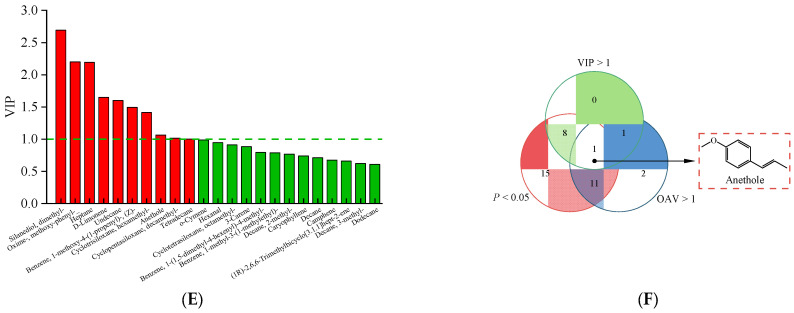
Effects of different reheating methods on the VOCs of CC: (**A**) represents a heatmap of the VOCs determined by GC−MS; (**B**) represents the types and numbers of the VOCs; (**C**) represents a score plot of OPLS−DA; (**D**) represents a cross-substitution plot of 200 permutations test; (**E**) represents a distribution of VIP values (red represents the characteristic flavors with VIP > 1, and green represents the characteristic flavors with VIP < 1); (**F**) represents key differential flavor compounds among the reheating treatments. CK was non-reheating treatment. WR was water-bath reheating. SR was steam reheating. AR was air−frying reheating. RR was roasting reheating. MR was microwave reheating.

According to Figure 8A and Table S1, a total of 41 VOCs were obtained in the CC by GC-MS. These compounds included 13 alkanes, 10 terpenes, 7 aromas, 3 alcohols, 2 aldehydes, and 6 other compounds. After the reheating process, 34 (WR), 34 (SR), 35 (AR), 33 (RR), and 36 (MR) VOCs were detected, higher than those detected in the CK group (Figure 8B). This indicated that reheating treatment could enrich the flavors of CC.

Alkanes typically had weak aromas and high flavor thresholds. They were generally used as carriers for other flavor substances, affecting the persistence and stability of overall flavors [53]. Terpenes had low flavor thresholds, which contributed greatly to the flavor of the CC [26]. The contents of D-limonene in the reheating treatments were 654.40 μg/kg (WR), 530.74 μg/kg (SR), 773.04 μg/kg (AR), 742.42 μg/kg (RR), and 767.35 μg/kg (MR) at a higher level. This compound could provide a lemony flavor and add a natural freshness to CC [54]. The content of trans-.alpha.-bergamotene was significantly increased (*p* < 0.05) after reheating, with the highest level in the AR samples. This indicated that AR treatment could enrich a woody flavor by increasing the content of trans-.alpha.-bergamotene. The contents of silanediol, dimethyl- were the highest in alcohols. Endo-borneol was produced after WR, SR, AR, RR, and MR treatments, with the contents of 15.16, 8.64, 9.02, 9.91, and 9.37 μg/kg, respectively. This result suggested that endo-borneol was an important alcohol compound in the CC after reheating, providing a fruity and milky flavor. The RR treatment had the highest content of total alcohols (567.96 μg/kg). In the RR process, a high-temperature reaction zone was formed by the metal pan, where the fat in the CC decomposed quickly and various VOCs were produced. These compounds could undergo complex reactions with each other, further contributing to the formation of aromas [37]. Aldehydes were mainly produced by the unsaturated fatty acid oxidation and Maillard reaction, with a low flavor threshold [55]. Hexanal exhibited a grassy flavor and nonanal exhibited a fatty flavor [56]. All treatments had no significant differences (*p* > 0.05) in hexanal. The contents of nonanal in the AR and RR samples were significantly higher than in the CK samples (*p* < 0.05), which was similar to the results of TBARS. This indicated that nonanal could effectively assess the degree of lipid oxidation and flavor deterioration in CC.

The OPLS-DA model is used to classify the VOCs of food in order to identify key differential flavor compounds [57]. Figure 8C illustrates that R^2^X (0.914), R^2^Y (0.926), and Q^2^ (0.706) were all nearly 1, showing that the model had good explanatory and predictive capabilities. The CK and SR samples were not separated well, indicating that SR treatment could not improve the flavor characteristics of CC. The AR and RR samples had similar features of VOCs because they were situated at the third quadrant. According to the results of the 200 permutations test, the intercept of R^2^ (0.498) and Q^2^ (−1.09) were below 0.5, suggesting that the model did not exhibit overfitting (Figure 8D). VIP indicates that the substance has a greater contribution to distinguishing among different groups in VOCs, and a VIP higher than 1 shows a greater contribution [58]. Figure 8E shows that the VIP values of 10 VOCs were more than 1, including silanediol, dimethyl-, oxime-, methoxy-phenyl-, heptane, D-limonene, undecane, benzene, 1-methoxy-4-(1-propenyl)-, (Z)-, cyclotrisiloxane, hexamethyl-, anethole, cyclopentasiloxane, ecamethyl-, and tetradecane. In addition, there were 35 VOCs corresponding to *p* < 0.05, and there were 15 VOCs corresponding to OAV > 1. Therefore, anethole was the only one that satisfied the three specified conditions (*p* < 0.05, OAV > 1, and VIP > 1) at the same time, considered as the key differential flavor-active compound in CC (Figure 8F).

## 4. Conclusions

In this study, the effects of reheating methods (WR, SR, AR, RR, and MR) on the physicochemical properties, oxidation, and flavor profiles of CC were evaluated. L* and b* had a slight change after the AR process, showing that AR could better preserve the original color of CC. The AR samples had the highest springiness, and the RR samples had the highest chewiness. The TBARS value of the AR treatment, as a high-temperature reheating method, showed a lower level. The RR samples had the highest degree of protein oxidation. In addition, the contents of umami-related amino acids were increased after the WR, AR, RR, and MR treatments, especially Glu. Glu and Cys were typically the taste-active amino acids in CC. According to the results of the E-nose and E-tongue, the AR samples had the highest response values of umami and richness. In addition, the AR treatment showed the greatest increase in the content of nitrogen oxides and alcohols. GC-MS analysis suggested that 41 VOCs were detected in CC. The contents of trans-.alpha.-bergamotene, nonanal, and copaene were significantly increased after AR treatment (*p* < 0.05). Endo-borneol was all produced after reheating. Anethole was regarded as the key differential flavor-active compound according to the OPLS-DA analysis. Overall, AR is the best reheating method in CC due to its better edible quality and richer flavors. The study provides a theoretical basis for choosing the appropriate reheating technologies for pre-cooked chicken products. Future research could explore how repeated reheating over time affects the nutritional content, sensory attributes, and shelf life of pre-cooked chicken products.

## Figures and Tables

**Table 1:** Sensors and their performance description of the PEN3 electronic nose.

Sensors	Performance Description
W1C	Sensitive to aromatic compounds (benzene)
W5S	Sensitive to nitrogen oxides
W3C	Sensitive to aromatic compounds (ammonia)
W6S	Sensitive to hydrides
W5C	Sensitive to aromatic compounds (short-chain alkane)
W1S	Sensitive to methyl compounds
W1W	Sensitive to inorganic sulfides
W2S	Sensitive to alcohols
W2W	Sensitive to aromatic compounds (organic sulfides)
W3S	Sensitive to long-chain alkanes

**Table 2:** Effects of different reheating methods on color of CC.

Reheating Methods	L*	a*	b*
CK	69.02 ± 1.34 ab	6.75 ± 0.26 c	26.51 ± 0.72 c
WR	66.31 ± 0.84 c	7.02 ± 0.12 b	29.14 ± 1.12 a
SR	70.28 ± 1.08 a	7.04 ± 0.14 b	27.28 ± 0.73 bc
AR	68.39 ± 0.92 b	7.18 ± 0.11 b	27.52 ± 0.49 bc
RR	64.15 ± 1.05 d	7.49 ± 0.17 a	28.01 ± 0.86 b
MR	63.45 ± 1.01 d	7.23 ± 0.14 b	27.68 ± 1.02 bc

Notes: CK was non−reheating treatment. WR was water−bath reheating. SR was steam reheating. AR was air−frying reheating. RR was roasting reheating. MR was microwave reheating. a–d mean significant differences between different reheating methods (*p* < 0.05).

## Data Availability

The original contributions presented in this study are included in the article/Supplementary Material. Further inquiries can be directed to the corresponding author.

## Supplementary Materials

The following supporting information can be downloaded at: https://www.mdpi.com/article/10.3390/foods14091574/s1, Table S1. Contents of volatile flavor compounds in CC with different reheating methods.
