# Supplementary material for: Impacts of Various Reheating Methods on Crispy Chicken: Physicochemical Properties, Oxidation and Flavor Profiles
Source: Foods. 2025 Apr 29;14(9):1574. doi: 10.3390/foods14091574 (PMC12072086; doi:10.3390/foods14091574)
Supplement: Supplementary file 1 [file foods-14-01574-s001.zip › foods-3544828-supplementary.pdf]

Table S1. Contents of volatile flavor compounds in CC with different reheating methods.

Name of volatile compound	CK (μg/kg)	WR (μg/kg)	SR (μg/kg)	AR (μg/kg)	RR (μg/kg)	MR (μg/kg)
Alkane						
Cycloheptasiloxane, tetradecamethyl-	11.28±2.55^{a}	13.03±1.93^{a}	8.99±1.26^{a}	11.41±4.85^{a}	10.64±0.52^{a}	11.38±1.61^{a}
Cyclohexasiloxane, dodecamethyl-	64.83±11.12^{a}	77.58±14.92^{a}	56.19±10.13^{a}	63.73±11.40^{a}	57.02±14.22^{a}	78.42±9.05^{a}
Cyclopentasiloxane, decamethyl-	75.19±16.22^{bc}	138.04±38.28^{a}	67.30±6.66^{c}	71.99±18.13^{c}	68.79±3.38^{c}	108.51±10.87^{ab}
Cyclotetrasiloxane, octamethyl-	124.33±18.17^{c}	155.55±6.90^{b}	122.43±10.41^{c}	131.36±22.04^{bc}	127.11±7.68^{c}	188.33±13.46^{a}
Cyclotrisiloxane, hexamethyl-	284.62±68.78^{bc}	365.32±35.77^{ab}	276.93±17.59^{bc}	320.53±88.12^{abc}	228.14±48.90^{c}	386.80±10.21^{a}
Decane	30.09±6.78^{cd}	59.46±10.25^{a}	39.23±2.58^{bc}	41.57±5.75^{b}	25.87±3.04^{d}	43.29±4.39^{b}
Decane, 2-methyl-	0.00±0.00^{d}	34.83±0.00^{a}	9.35±0.26^{b}	0.00±0.00^{d}	7.20±1.47^{c}	9.42±0.43^{b}
Decane, 3-methyl-	13.27±3.18^{c}	32.05±3.32^{a}	17.73±2.08^{b}	19.18±1.44^{b}	0.00±0.00^{d}	29.91±2.87^{a}
Decane, 5-methyl-	3.28±0.61^{c}	10.03±1.41^{a}	6.26±0.32^{b}	5.19±0.38^{b}	3.73±0.88^{c}	5.74±0.62^{b}
Dodecane	27.33±5.97^{b}	45.07±5.96^{a}	21.01±2.63^{b}	28.24±8.87^{b}	30.82±0.04^{b}	29.68±2.73^{b}
Heptane	246.90±155.67^{a}	156.78±2.91^{ab}	78.77±2.15^{c}	153.55±9.90^{ab}	50.43±15.17^{c}	241.90±18.39^{a}
Tetradecane	63.73±7.00^{a}	48.47±6.21^{b}	14.03±8.97^{c}	16.37±2.88^{c}	14.23±0.77^{c}	20.35±0.20^{c}
Undecane	0.00±0.00^{c}	158.32±18.12^{a}	54.97±3.96^{b}	54.53±10.94^{b}	0.00±0.00^{c}	68.77±5.75^{b}
Terpene						
.alpha.-Pinene	0.00±0.00^{c}	19.40±1.69^{ab}	0.00±0.00^{c}	16.40±2.53^{b}	22.66±7.05^{a}	24.31±2.78^{a}
.beta.-Bisabolene	5.89±2.30^{bc}	5.96±0.88^{bc}	2.87±1.05^{c}	6.71±3.71^{ab}	9.62±0.88^{a}	4.61±0.74^{bc}
.beta.-Pinene	21.89±2.96^{a}	17.45±1.27^{b}	18.68±2.86^{b}	0.00±0.00^{c}	0.00±0.00^{c}	0.00±0.00^{c}

Name of volatile compound	CK (μg/kg)	WR (μg/kg)	SR (μg/kg)	AR (μg/kg)	RR (μg/kg)	MR (μg/kg)
3-Carene	107.20± 25.85[abc]	127.69± 12.56[a]	96.21± 12.68[bc]	84.15± 16.79[c]	98.20± 3.90[bc]	125.07± 10.81[ab]
Camphene	16.96± 4.59[c]	14.38± 1.23[c]	21.26± 7.02[c]	24.34± 3.13[bc]	31.74± 6.05[ab]	36.56± 7.39[a]
Caryophyllene	14.96± 10.66[b]	33.34± 5.63[a]	1.63± 0.07[c]	23.92± 13.06[ab]	30.55± 0.98[a]	23.66± 2.05[ab]
Copaene	8.29± 1.43[c]	12.77± 1.33[ab]	0.00± 0.00[d]	14.10± 3.78[a]	12.83± 2.23[ab]	9.65± 0.48[bc]
D-Limonene	524.70± 71.79[a]	654.40± 194.35[a]	530.74± 114.69[a]	773.04± 106.83[a]	742.42± 211.76[a]	767.35± 59.95[a]
Longifolene	0.00± 0.00[b]	0.00± 0.00[b]	0.00± 0.00[b]	5.28± 1.35[a]	0.00± 0.00[b]	5.99± 2.50[a]
trans-.alpha.-Bergamotene	0.00± 0.00[d]	4.16± 0.78[bc]	2.46± 0.33[c]	6.18± 1.02[a]	4.63± 1.21[ab]	4.11± 1.34[bc]
Alcohol						
endo-Borneol	0.00± 0.00[c]	5.16± 2.82[b]	8.64± 0.08[a]	9.02± 3.07[a]	9.91± 1.57[a]	9.37± 0.16[a]
Silanediol, dimethyl-	505.01± 103.81[b]	646.09± 57.72[b]	492.77± 78.30[b]	466.72± 49.48[bc]	289.45± 154.32[c]	922.43± 154.01[a]
Terpinen-4-ol	10.16± 2.25[ab]	13.77± 2.49[a]	8.76± 0.53[b]	8.31± 3.24[b]	11.44± 2.49[ab]	8.63± 0.21[b]
Aroma						
Anethole	36.36± 21.01[c]	77.20± 0.11[a]	6.56± 0.05[d]	0.00± 0.00[d]	5.67± 3.36[d]	58.03± 3.24[b]
Benzene, 1-(1,5-dimethyl-4-hexenyl)-4-methyl-	38.85± 15.22[bc]	51.24± 6.57[ab]	28.73± 6.58[c]	31.35± 10.01[c]	59.65± 6.21[a]	36.24± 3.12[bc]
Benzene, 1-methoxy-4-(1-propenyl)-, (Z)-	0.00± 0.00[d]	0.00± 0.00[d]	22.45± 5.55[c]	93.48± 22.08[a]	0.00± 0.00[d]	48.54± 0.60[b]
Benzene, 1-methyl-3-(1-methylethyl)-	0.00± 0.00[c]	0.00± 0.00[c]	0.00± 0.00[c]	37.66± 1.15[b]	43.18± 2.13[a]	0.00± 0.00[c]
Estragole	0.00± 0.00[b]	0.00± 0.00[b]	0.00± 0.00[b]	7.57± 2.54[a]	0.00± 0.00[b]	7.82± 0.09[a]
o-Cymene	62.13± 6.10[a]	0.00± 0.00[c]	46.86± 7.01[b]	0.00± 0.00[c]	0.00± 0.00[c]	0.00± 0.00[c]

Name of volatile compound	CK (μg/kg)	WR (μg/kg)	SR (μg/kg)	AR (μg/kg)	RR (μg/kg)	MR (μg/kg)
Oxime-, methoxy-phenyl-_	93.63± 19.59[d]	134.55± 46.86[cd]	73.42± 58.23[d]	270.14± 49.72[b]	459.47± 81.25[a]	201.01± 25.94[bc]
Aldehyde						
Hexanal	311.00± 27.59[a]	314.30± 56.16[a]	322.15± 89.71[a]	378.83± 21.41[a]	402.55± 34.45[a]	335.88± 116.22[a]
Nonanal	52.46± 9.72[b]	55.82± 11.59[b]	55.1± 10.12[b]	76.13± 3.40[a]	76.65± 17.3[a]	68.59± 4.98[ab]
Others						
(1R)-2,6,6-Trimethylbicyclo[3.1.1]hept-2-ene	17.28± 4.85[b]	0.00± 0.00[d]	17.32± 2.07[b]	19.67± 0.66[b]	9.77± 0.49[c]	23.97± 1.72[a]
1,3-Cyclohexadiene,5-(1,5-dimethyl-4-hexenyl)-2-methyl-, [S-(R*,S*)]-	0.00± 0.00[c]	15.39± 1.22[a]	0.00± 0.00[c]	0.00± 0.00[c]	0.00± 0.00[c]	10.74± 1.11[b]
Cyclohexene, 1-methyl-4-(1-methylethylidene)-	0.00± 0.00[c]	5.47± 0.70[b]	0.00± 0.00[c]	7.76± 0.92[a]	6.96± 1.38[a]	0.00± 0.00[c]
Cyclohexene, 3-(1,5-dimethyl-4-hexenyl)-6-methylene-, [S-(R*,S*)]-	0.00± 0.00[c]	0.00± 0.00[c]	5.23± 1.17[b]	0.00± 0.00[c]	18.67± 1.01[a]	0.00± 0.00[c]
Fenchone	14.51± 2.63[b]	24.97± 4.13[a]	13.38± 0.99[b]	10.91± 0.69[b]	12.41± 0.75[b]	14.49± 0.36[b]
Tricyclo[2.2.1.0(2,6)]heptane, 1,7-dimethyl-7-(4-methyl-3-pentenyl)-, (-)-	6.75± 1.69[ab]	7.88± 1.38[a]	4.15± 0.83[b]	6.42± 3.10[ab]	7.05± 0.66[ab]	5.87± 1.18[ab]

Notes: CK was non-reheating treatment. WR was water bath reheating. SR was steam reheating. AR was air frying reheating. RR was roasting reheating. MR was microwave reheating. Results are expressed as mean values of three determinations ± SD. Different letters in the same row show the significant difference ($P < 0.05$).
